# New insights into *Chlamydia* pathogenesis: Role of leukemia inhibitory factor

**DOI:** 10.3389/fcimb.2022.1029178

**Published:** 2022-10-18

**Authors:** Jun Wang, Katherine Wang

**Affiliations:** ^1^ Canadian Center for Vaccinology, Halifax, NS, Canada; ^2^ Department of Microbiology & Immunology, Halifax, NS, Canada; ^3^ Department of Pediatrics, Faculty of Medicine, Dalhousie University, Halifax, NS, Canada; ^4^ Izaak Walton Killam (IWK) Health Centre, Halifax, NS, Canada

**Keywords:** *Chlamydia*, leukemia inhibitory factor (LIF), ectopic pregnancy, infertility, cervical cancer, ovarian cancer, pelvic inflammatory disease (PID)

## Abstract

*Chlamydia trachomatis (Ct)* is the leading cause of bacterial sexually transmitted infections worldwide. Since the symptoms of *Ct* infection are often subtle or absent, most people are unaware of their infection until they are tested or develop severe complications such as infertility. It is believed that the primary culprit of *Ct*-associated tissue damage is unresolved chronic inflammation, resulting in aberrant production of cytokines, chemokines, and growth factors, as well as dysregulated tissue influx of innate and adaptive immune cells. A member of the IL-6 cytokine family, leukemia inhibitory factor (LIF), is one of the cytokines induced by *Ct* infection but its role in *Ct* pathogenesis is unclear. In this article, we review the biology of LIF and LIF receptor (LIFR)-mediated signaling pathways, summarize the physiological role of LIF in the reproductive system, and discuss the impact of LIF in chronic inflammatory conditions and its implication in *Ct* pathogenesis. Under normal circumstances, LIF is produced to maintain epithelial homeostasis and tissue repair, including the aftermath of *Ct* infection. However, LIF/LIFR-mediated signaling – particularly prolonged strong signaling – can gradually transform the microenvironment of the fallopian tube by altering the fate of epithelial cells and the cellular composition of epithelium. This harmful transformation of epithelium may be a key process that leads to an enhanced risk of infertility, ectopic pregnancy and cancer following *Ct* infection.

## Introduction

The *Chlamydiae* family is a diverse group of obligatory intracellular bacteria, comprised of both pathogens and commensals, and found in varying habitats that can extend as far as the bottom of the Arctic Ocean (Dharamshi et al., 2020). *Chlamydia trachomatis* (*Ct*) is a human pathogen and the most common bacterial cause of sexually transmitted infections worldwide (WHO, 2021). An uptick in the reporting of chlamydial urogenital infections began in the 1990s following improvements in diagnostic technologies, and the rates have been increasing annually ever since (CDC, 2020; Huai et al., 2020; Control ECfDPa, 2022). *Ct* affects mostly young women aged 15-24, but can infect both men and women of all age groups (CDC, 2020; Huai et al., 2020; Control ECfDPa, 2022). Urogenital *Ct* infection is clinically associated with cervicitis, urethritis, endometritis, and salpingitis in women, and urethritis, proctitis and epididymitis in men (CDC, 2020; Control ECfDPa, 2022). However, *Ct* urogenital infections are notorious for being asymptomatic or sub-clinically symptomatic in as many as 70% of women and 50% of men (CDC, 2020; Gupta et al., 2021; Control ECfDPa, 2022). These “silent” *Ct* infections are often left untreated, which can last for months to years (McCormack et al., 1979; Morré et al., 2002; Molano et al., 2005; Geisler, 2010). Approximately 17% of *Ct*-infected women go on to develop pelvic inflammatory disease (PID) and serious complications such as tubal factor infertility (TFI), ectopic pregnancy and chronic pelvic pain (Cherpes et al., 2011; Brunham et al., 2015; Price et al., 2016). Repeated *Ct* infections and recurrent PID episodes are associated with a greater risk for adverse reproductive outcomes (Hillis et al., 1997; Hosenfeld et al., 2009; Batteiger et al., 2010; Trent et al., 2011; Bautista et al., 2018). Epidemiological evidence indicate that *Ct* infections significantly increase the risk of cervical (Zhu et al., 2016) and ovarian cancer (Hosseininasab-Nodoushan et al., 2021), presumably due to unresolved chronic inflammation (Clendenen et al., 2011; Peres et al., 2021). While considerable efforts have been made in attempts to elucidate *Ct* pathogenesis, there are still gaps regarding the definitive molecular and cellular mechanisms that cause *Ct*-associated tissue damage.

*Chlamydia* species have a unique biphasic lifecycle consisting of an extracellular form of non-replicative but infectious elementary body (EB), and an intracellular form of replicative reticulate body (RB) (Moulder, 1991; Hackstadt et al., 1997). Infection is normally initiated at the single layer of mucosal epithelium in the lower genital tract, followed by bacterial ascension to the upper genital tract. Once inside the host cells, the non-replicative EB resides within a cytoplasmic inclusion body, where it differentiates into a replicative RB which then multiplies *via* binary fission. As an inclusion fills with progeny, RBs transform back into infectious EBs that are released from host cells to infect other neighboring cells (Moulder, 1991; Hackstadt et al., 1997). Immediately after intracellular infection, cell-autonomous immunity and innate defence mechanisms are triggered to fight against intracellular *Ct* infections (Mostowy and Shenoy, 2015; Elwell et al., 2016). These initial molecular and cellular events are followed by induction of *Ct*-specific adaptive humoral and cellular immune responses that are required for the ultimate elimination of intracellular *Ct* infection (Kelly, 2003; Batteiger et al., 2010; Helble and Starnbach, 2021). Notably, the inflammatory responses associated with innate and adaptive immunity are also involved in the tissue repair and regeneration processes (Eming et al., 2017), which are critical for removing the cellular debris from the infected tissue site, maintaining the tissue homeostasis, and restoring the normal structure and physiological integrity of the organ after infection (Gurtner et al., 2008). Of note, *Chlamydia* species are known for their strong ability to exploit various strategies to enhance intracellular survival (Fischer and Rudel, 2018; Chen et al., 2019). Some RBs ultimately differentiate into a morphologically distinct persistence form called an aberrant body (AB) in response to host defense molecules and certain antibiotic treatments (Phillips et al., 1984; Mazzoli et al., 2000; Hogan et al., 2004). While ABs are nonreplicative, they remain dormant inside host cells for a long period of time and are capable of reverting back to replicative RBs when more favorable conditions are present, such as the use of an immune suppressant (Yang et al., 1983; Beatty et al., 1994; Laitinen et al., 1996). As such, the host responses to *Ct* infection are complex and often modified by persistent infection, reactivation, and/or repeated exposures (Schuchardt and Rupp, 2018). The most widely accepted theory of *Ct* pathogenesis is that unresolved chronic inflammation is the primary culprit of *Ct*-associated tissue damage. This involves aberrant production of cytokines, chemokines, and growth factors, as well as the influx of innate and adaptive immune cells that collectively trigger aggravated wound healing and tissue repair processes (Karin and Clevers, 2016). While numerous cytokines have been implicated as part of the host response to *Ct* infection (Yang, 2003; Xiang et al., 2021), leukemia inhibitory factor (LIF), a member of the interleukin 6 (IL-6) cytokine family, has been largely ignored. Recent studies indicate that LIF is readily induced by *Ct* infection (Hess et al., 2001; Kessler et al., 2019), and that increased LIF expression is often linked to *Ct*-associated ectopic pregnancies in humans (Refaat et al., 2016) as well as severe oviductal pathology in mice (Hou et al., 2018). Therefore, LIF likely holds new perspectives of *Ct* pathogenesis. In this article, we review the biology of LIF and LIF receptor (LIFR)-mediated signaling pathways, summarize the physiological role of LIF in the reproductive system, and discuss the impact of LIF in chronic inflammatory conditions and its implication in *Ct* pathogenesis.

## The biology of LIF and LIF receptors

**Figure 1:**
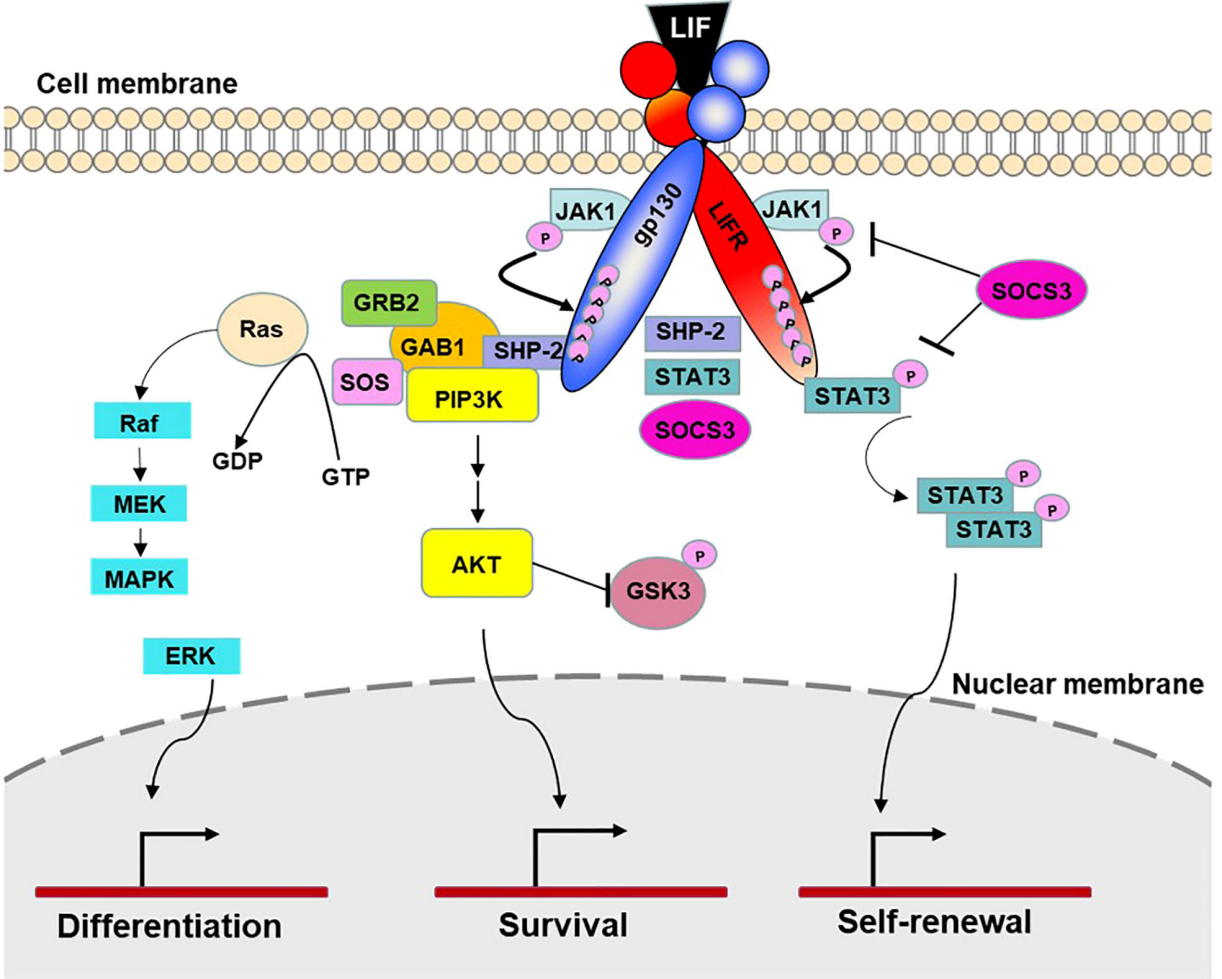
LIF signaling. LIF signaling occurs through a receptor complex of gp130 and LIFR. Under resting conditions, both receptor chains are constitutively associated with inactivated JAK1 molecules. Upon LIF stimulation, LIF binds to LIFR and gp130 and initiates signal transduction by transactivation of the gp130-bound and LIFR-bound JAK1 molecules. JAK1 then phosphorylates five tyrosine molecules on each receptor chain, which create the docking sites for the transcription factors STAT3 and SHP-2, leading to activation of JAK/STAT3, MAPK and PI3K/AKT signaling pathways. The docking site also recruits transcription factor SOCS3, which is a predominant negative pathway that inhibits JAK-STAT3 activation. By controlling cellular differentiation, survival and self-renewal, these pathways collectively contribute to specific physiological and immunological processes such as tissue homeostasis, hematopoiesis, pregnancy, bone remodeling, neuromuscular system, inflammatory conditions, and cancer (Nicola et al., 2015 and Rose-John et al., 2018).

**Figure 2:**
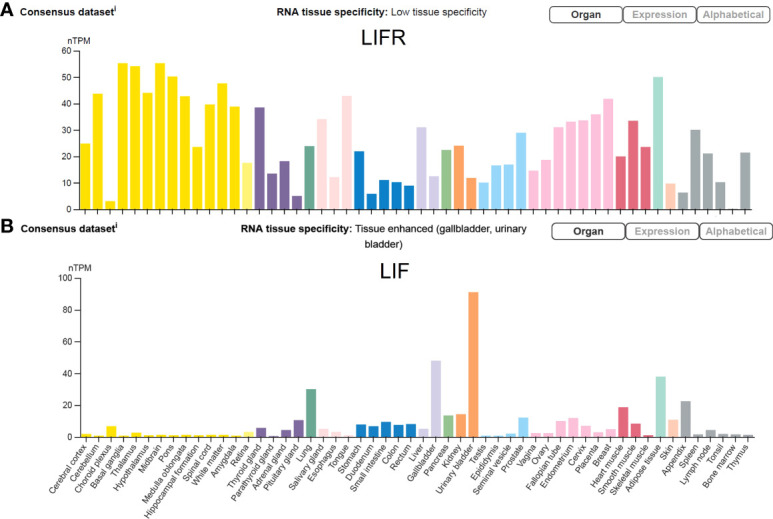
LIFR and LIF expression in human tissues. The transcripts of LIFR and LIF in 55 healthy human tissues are displayed at normalized expression levels (nTPM) that are created by combining transcriptomics datasets of The Human Protein Atlas (HPA) project and The Genotype-Tissue Expression (GTEx) project. Figure is available from The Human Protein Atlas website (accessed on August 15, 2022).

LIF is encoded by a single copy gene on chromosome 22 in humans and chromosome 11 in mice (Gough et al., 1988; Metcalfe, 2011). The coding region is highly conserved among several mammalian species with more than 75% homology (Willson et al., 1992). LIF belongs to the IL-6 cytokine family that consists of IL-6, IL-11, IL-27, LIF, oncostatin M (OSM), ciliary neurotrophic factor (CNTF), cardiotrophin 1 (CT-1) and cardiotrophin-like cytokine factor 1 (CLCF1) (Nicola and Babon, 2015; Rose-John, 2018). All IL-6 family members utilize a receptor complex that is comprised of the shared signal-transducing receptor β-subunit gp130 (also known as IL-6Rβ), together with either a ligand-binding non-signaling receptor α-subunit and/or a signaling receptor β-subunit resembling gp130 for signal transduction. The specific composition of different ligand-receptor complexes underscores both redundant and unique biological activities of IL-6 family cytokines (Rose-John, 2018). In the case of LIF signaling, LIF binds to a receptor complex consisting of gp130 and LIFR, both of which are constitutively associated with receptor-associated JAK molecules, particularly JAK1 (Rodig et al., 1998; Chung et al., 2006; Takahashi et al., 2008), and initiates the signal transduction cascades that lead to activation of JAK/STAT3, MAPK and PI3K/AKT signaling pathways (Nicola and Babon, 2015). Notably, JAK/STAT3 signaling also induces the expression of suppressor of cytokine signaling 3 (SOCS3) (Babon et al., 2014), which binds to both JAK1 and gp130 and induces their ubiquitination and degradation, thereby shutting down entire signaling cascades (Kershaw et al., 2013) (**Figure 1**). While these pathways collectively contribute to cellular differentiation, survival and self-renewal, the qualitative and quantitative contributions of individual pathways are cell-type specific, leading to the pleiotropic effects of LIF (Nicola and Babon, 2015). Although named for its ability to inhibit proliferation of a myeloid leukemia cell line by inducing its terminal differentiation into macrophages (Gearing et al., 1987), LIF also regulates the growth and differentiation of embryonic stem cells (Smith et al., 1988; Williams et al., 1988), peripheral neurons (Murphy et al., 1991; Li et al., 1995; Cheng and Patterson, 1997), osteoblasts (Matsushita et al., 2014; Sims, 2020), adipocytes (Guo et al., 2021; Zeng et al., 2022), hepatocytes (Baumann and Wong, 1989), and endothelial cells (Ferrara et al., 1992; Li et al., 2022). This multifarious property of LIF has led to several rediscoveries of the protein and a variety of synonyms have been used in the older literature (Alexander et al., 1994; Nicola and Babon, 2015) (**Table 1**). Since its original discovery, LIF has been shown to display a diverse range of biological activities in embryo implantation, bone metabolism and remodelling, immune regulation, as well as the development of uterine, hematopoietic, and nervous systems (Kurzrock et al., 1991; Mathieu et al., 2012; Nicola and Babon, 2015). The broad effects of LIF also coincide with ubiquitous expression of LIFR in The Human Protein Atlas (Human Protein Atlas, 2022) (**Figure 2A**). However, contrary to the LIFR expression pattern, LIF expression is enriched in glandular and luminal cells in select healthy human tissues (Human Protein Atlas, 2022) (**Figure 2B**). Overall, LIF production can be detected in many cell types, such as stromal fibroblasts (Tomida et al., 1984), bone marrow stromal cells (Wetzler et al., 1994), activated monocytes and macrophages (Anegon et al., 1990), astrocytes (Wesselingh et al., 1990), and T cells (Gearing et al., 1987).

**Table 1:** Synonyms for LIF.

Abbreviation	Name
LIF	Leukemia inhibitory factor
D-factor	Differentiation-inducing factor
DIF	Differentiation-inducing factor
DIA	Differentiation inhibitory activity
DRF	Differentiation-retarding factor
MLPLI	Melanoma-derived lipoprotein lipase inhibitor
HILDA	Human interleukin for DA-1 cells
CDF	Cholinergic differentiation factor
OAL	Osteoclast-activating factor
HSF-III	Hepatocyte-stimulating factor III

## The role of LIF/LIFR in the female reproductive system

Menstruation is a key feature of the mammalian female reproductive system that is characterized by monthly remolding, shedding and regeneration of the endometrium under the influence of ovarian hormones and divided into an estrogen (E2)-dominated proliferative phase and a progesterone (P4)-dominated secretory phase (Reed and Carr, 2000). Cell types in the human endometrium include epithelial cells, stromal fibroblasts, immune cells, vascular endothelium, vascular smooth muscle cells and stem/progenitor cells. The epithelial cells are further divided into three subpopulations: luminal epithelium, glandular epithelium, and ciliated epithelial cells (Wang et al., 2020; Giudice, 2020). Functionally, the glandular and luminal epithelia are required for embryo attachment whereas the ciliated epithelial cells are needed to facilitate sperm transport, fluid movement and removal of debris from the uterine cavity (Giudice, 2020). During the menstrual cycle, the human endometrium undergoes a decidualization process, which is characterized by the transformation of stromal cells to round epithelioid cells capable of secreting growth hormones (e.g., prolactin and insulin-like growth factor binding protein-1), an influx of specialized uterine natural killer cells, and vascular remodeling to support the maternal blood supply to the growing embryo (Okada et al., 2018). The decidua plays a critical role in regulating trophoblast invasion, modulating the local immune response at the fetal-maternal interface, and development of the placenta (Ramathal et al., 2010; Okada et al., 2018). The human decidua is formed routinely and is shed off in the absence of an embryo in the endometrium, whereas the decidua is only formed upon embryo implantation in mice (Ramathal et al., 2010). While the steroid hormones E2 and P4 have paramount effects on the development and differentiation of decidua, the hormone effects are also mediated either directly or indirectly through locally produced growth factors and cytokines (Large and DeMayo, 2012). LIF is one of the most important cytokines essential for decidualization, embryo implantation, and regulation of immune tolerance at the fetal-maternal interface (Stewart et al., 1992; Aghajanova, 2004; Kimber, 2005).

**Figure 3:**
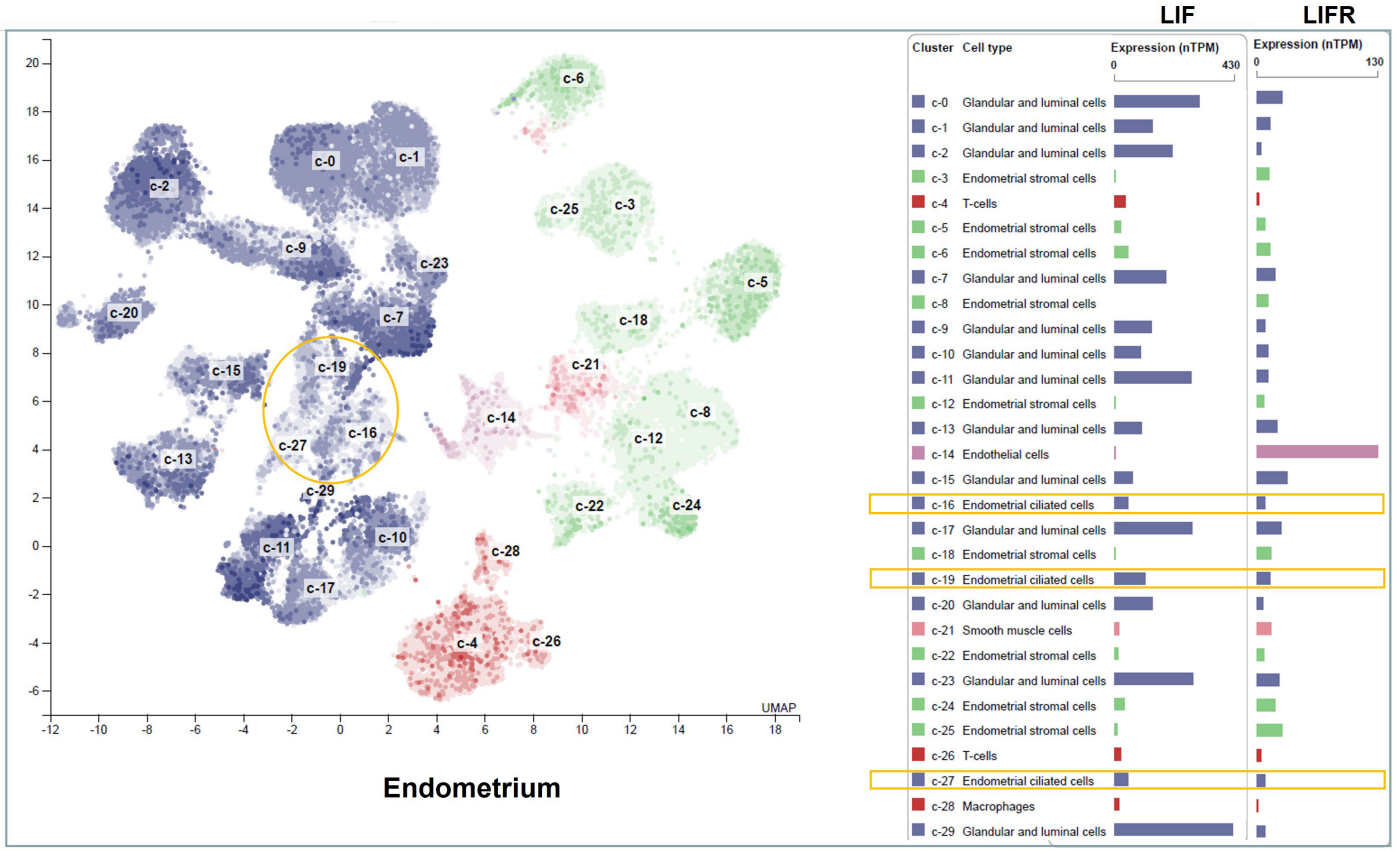
LIF and LIFR mRNA expression in different cell types of the human endometrium. The transcripts of LIF and LIFR in healthy human endometrial tissues are displayed at normalized expression levels (nTPM). Cell types in the endometrium include epithelial cells (blue), stromal fibroblasts (green), immune cells (red), vascular endothelium (purple), and vascular smooth muscle cells (orange). The epithelial cells are further divided into luminal epithelium, glandular epithelium and ciliated epithelial cells. The ciliated epithelial cell clusters are highlighted in yellow boxes. Figure is available from The Human Protein Atlas website (accessed on August 15, 2022).

In healthy women, LIF mRNA and protein are expressed by the endometrium throughout the menstrual cycle, with a striking increase in the mid-secretory to late-secretory phase (Charnock-Jones et al., 1994; Arici et al., 1995; Vogiagis et al., 1996) which is a finite period defined as the implantation window (Harper, 1992). Single-cell transcriptomic analysis of the human endometrium during the menstrual cycle has revealed an abrupt and strong transcriptomic activation in epithelial cells, and a widespread decidualization feature in the stromal cells during the implantation window (Wang et al., 2020). LIF mRNA is most abundantly expressed in the glandular and luminal epithelial cells (Human Protein Atlas, 2022) (**Figure 3**), and the same expression pattern is detected by immunohistochemistry staining (Charnock-Jones et al., 1994; Vogiagis et al., 1996). In parallel with the pattern of LIF expression, LIFR also peaks in human endometrium during the mid-secretory phase (Charnock-Jones et al., 1994; Cullinan et al., 1996), with strong expression in endothelial cells, intermediate levels in glandular epithelial cells, luminal epithelial cells and smooth muscle cells, but very low levels in immune cells (Human Protein Atlas, 2022) (**Figure 3**). While the tumor suppressor gene p53 is required to maintain both basal and inducible transcription of LIF in the uterus (Hu et al., 2007), endometrial expression of LIF and LIFR are induced by E2 (Chen et al., 2000; Ding et al., 2008; Liang et al., 2014; Yoo et al., 2019; Polim et al., 2022) and P4 (Ding et al., 2008; Yoo et al., 2019), respectively. Interestingly, P4 also induces LIF expression in bovine endometrium epithelial cells *in vitro (* Feng et al., 2022). As such, the concerted physiological effects of E2 and P4 in the reproductive system are executed, if not entirely, *via* regulation of LIF/LIFR signaling pathways, particularly the JAK-STAT3 pathway (Cheng et al., 2001). LIF activates over 40 different transcription factors that control a multitude of physiological pathways and signal cascades for epithelial polarity and apoptosis, epithelial-mesenchymal interaction, angiogenesis, stromal decidualization and cell proliferation, creating a favorable environment in the uterus to promote embryo implantation (Rosario et al., 2014; Salleh and Giribabu, 2014; Rosario and Stewart, 2016).

Functional LIFR and gp130 are expressed on both luminal and granular epithelial cells, human oocytes and preimplantation embryos (van Eijk et al., 1996). This expression pattern suggests that LIF/LIFR-mediated signaling pathways may promote embryo implantation *via* both autocrine and paracrine pathways. Several studies using gene-deficient mice and embryo-transfer approaches indicate that maternal LIF/LIFR-mediated signaling pathways are essential in embryo implantation (Stewart et al., 1992; Cheng et al., 2002; Cheng et al., 2017). Both LIF-deficient mice (Stewart et al., 1992) and mice with LIFR-deficiency specifically in luminal and glandular epithelial cells (Cheng et al., 2017), fail to support embryo implantation and development. However, the development of LIF-deficient embryos in LIF-deficient mice can be rescued by recombinant LIF injections (Chen et al., 2000). In accordance with the animal studies, reduced LIF production is observed in women with unexplained infertility and recurrent miscarriages compared to healthy fertile women in many studies (Laird et al., 1997; Hambartsoumian, 1998; Wu et al., 2013; Margioula-Siarkou et al., 2016; Margioula-Siarkou et al., 2017; Alzaidi et al., 2021), although there are exceptions (Olivennes et al., 2003; Xu et al., 2012; Karaer et al., 2014). Interestingly, certain LIF polymorphisms have been associated with recurrent implantation failure (Vagnini et al., 2019) and women with LIF mutations also have poor success rates of implantation and fertilization (Giess et al., 1999; Novotný et al., 2009) (**Table 2**).

**Table 2:** Influence of LIF in human infertility, miscarriage and assisted pregnancy Brief description of the study.

Normal fertile women, women with unexplained infertility and women who suffered recurrent miscarriages were studied. Researchers obtained uterine flushings from all the women for analysis. LIF in flushings obtained from women with unexplained infertility was significantly lower than normal fertile women. They concluded that decreased concentrations of LIF in women with unexplained infertility indicate how important the cytokine is to embryo implantation.	Laird et al., 1997
32 women with unexplained infertility and 17 fertile women were studied. Endometrial biopsy samples were obtained and studied using a sensitive enzyme-linked immunosorbent assay (ELISA). LIF secretion was 2.2 times higher during the secretory phase than proliferative phase in fertile women whereas infertile women did not have this elevation in cytokine production. They concluded that the deregulation of endometrial LIF secretion may be linked to unexplained infertility and repetitive failures of implantation.	Hambartsoumian, 1998
30 infertile women with multiple implantation failures (MIF) and a fertile control group were studied. Researchers obtained endometrial biopsies in the proliferative phase and measured expression of LIF using immunohistochemistry and western blotting. Lower expression of LIF was found in infertile women with MIF compared to fertile women. They determined that initial lower expression of LIF during the proliferative phase may be a cause of multiple failures of implantation.	Wu et al., 2013
Women diagnosed with infertility and a control group of fertile women were studied. Researchers took an endometrial biopsy post ovulation to examine LIF and LIF-R expression. No significant differences in LIF/LIF-R was found in the stromal cells but there was a significant reduction in LIF/LIF-R expression in infertile women in epithelial cells. They concluded that LIF and LIF-R are significantly under expressed in epithelial cells of infertile women.	Margioula-Siarkou et al., 2017
75 infertile women and 40 control women were studied. Levels of LIF and IL-11 were examined using qRT-PCR. Lower levels of LIF and IL-11 were linked to increased risk of having PCOS, tubal factor, and unexplained fertility, likely due to the critical role these genes play in embryo implantation.	Alzaidi et al., 2021
30 women with idiopathic recurrent pregnancy loss (RPL) and 30 fertile controls were studied. Endometrial biopsies were used to evaluate PROK1 and LIF expression. PROK1 and LIF expression was significantly increased in the endometriums of women with idiopathic RPL. They concluded that increased mRNA expression of PROK1 and LIF could contribute to risk of RPL.	Karaer et al., 2014
148 IVF patients received uterine flushing during egg retrieval to assess LIF levels. Uterine flushing did not appear to affect pregnancy rates. LIF was found in 46% of patients at time of sample collection but no indication of better pregnancy rates in patients that had LIF compared to those who did not.	Olivennes et al., 2003
Women who were undergoing assisted reproduction were studied. Researchers obtained endometrial biopsies from women with recurrent pregnancy loss and a control group of fertile women, 7 days after luteinizing hormone peaked and LIF expression was examined. There was no significant difference in LIF expression between the two study groups.	Xu et al., 2012
44 women with recurrent implantation failure (RIF), 63 women who had children *via* IVF and 65 fertile women with children and no history of miscarriage were studied. Women who had a polymorphism in ESR1 and LIF had increased chances of presenting with RIF. They concluded that ER1 and LIF polymorphisms can predict RIF.	Vagnini et al., 2019
Women from 4 different groups of diagnoses associated with LIF mutations and various causes of infertility were studied to determine the impact of mutation in the LIF gene on assisted pregnancy *via in vitro* fertilization (IVF). They concluded that women with LIF mutations, infertility and endometriosis have poorer outcomes with IVF than other groups.	Novotný et al., 2009
Nulligravid infertile women, a fertile control group and unrelated control group were screened for LIF gene mutations. 3 point-mutations were identified in the infertile group that reduced biological activity of the LIF protein. They concluded that a heterozygous LIF mutation could result in decreased availability of LIF in the uterus, leading to implantation failure and thus infertility.	Giess et al., 1999

Although the collective evidence support a critical role for LIF in promoting embryo implantation, recombinant human LIF did not yield any beneficial effects in one randomized, double-blind, placebo-controlled, multicenter study (Brinsden et al., 2009). Surprisingly, recombinant LIF, administered to infertile women subcutaneously for 7 days starting on the day of embryo transfer, significantly reduced clinical pregnancy rates (Brinsden et al., 2009). Although the reason behind this unexpected result remains to be investigated, it is possible that a balanced LIF/LIFR signal is critical for successful pregnancies; both too little and too much LIF production can cause infertility. A prolonged strong LIF signal may trigger activation of a negative feedback pathway mediated by SOCS, subsequently promoting degradation of receptors, leading to implantation failure (Carvalho et al., 2014; Zhao et al., 2021). Furthermore, implantation is a complex and highly organized process that involves active crosstalk between a receptive uterus and a competent blastocyte in a time- and location-specific manner (Kim and Kim, 2017). Thus, dysregulation of LIF/LIFR may lead to ectopic pregnancy and potentially other fertility-related issues (Krishnan et al., 2013).

## The potential impact of LIF/LIFR axis in *Ct* pathogenesis

In addition to its physiological roles, the LIF/LIFR axis has been broadly discussed in the context of chronic inflammation, including chronic airway inflammation (Knight, 2001), cutaneous inflammation (Zhu et al., 2001), neuroinflammation (Linker et al., 2008; Pan et al., 2008), and cancer-associated inflammation (Christianson et al., 2021), mostly as an anti-inflammatory cytokine. In infection models, LIF is recognized as a vital stem cell growth factor that protects the lung from collateral damage during inflammatory attack against viral infections (Quinton et al., 2012; Foronjy et al., 2014).

The production of LIF during chlamydial infection has been reported by several *in vitro* and *in vivo* studies (Hess et al., 2001; Peters et al., 2005; Refaat et al., 2016; Hou et al., 2018; Kessler et al., 2019). It was first identified through comparing the transcriptomic profile of 1176 genes in DNA arrays of *Ct*-infected and mock-infected epithelial HeLa cells, in which 18 genes, including LIF, were up-regulated by *Ct* infection (Hess et al., 2001). This observation has been recently confirmed in human fallopian tube organoid cultures (Kessler et al., 2019), which provide a novel *in vitro* system for recapitulating *Ct* infection *in vivo*. It was demonstrated that LIF is readily induced by *Ct* infection, along with robust activation of type I interferon (IFN-β) signaling and upregulation of inducible nitric oxide synthase (NOS2) to control *Ct* replication in organoids. In addition, LIF/LIFR signaling is involved in tissue injury and repair responses that are needed for maintaining epithelial homeostasis and organoid renewal (Kessler et al., 2019). Therefore, LIF seems to be part of cell-autonomous immunity, and its production is a protective response. Notably, LIF production is only triggered in mice by a pathological, plasmid-containing *Chlamydia muridarum*, but not a plasmid-free one. The level of LIF is closely associated with the degree of bacterial ascension in the upper genital tract and the formation of hydrosalpinx (Hou et al., 2018), a common tissue pathology associated with *Ct* infection in both humans and mice that can cause infertility. Similarly, LIF mRNA and protein are most detected at high levels in human fallopian tube samples obtained from ectopic pregnancies associated with *Ct* infections but not non-*Ct* infections (Refaat et al., 2016). While these experimental results support a potential role of LIF in *Ct* pathogenesis, it is intriguing why a protective response is highly associated with tissue pathology.

The human fallopian tube is a conduit that has a major functional role in oocyte pickup, fertilization, and embryo transport. The fallopian tube mucosa contains two major histologic cell types: ciliated epithelial cells and secretory epithelial cells, which work together for effective tubal transport of ova, sperm, and embryos for successful spontaneous pregnancy (Lyons et al., 2006). Propulsion of gametes and embryos is achieved by complex interactions between muscle contractions, ciliary activity, and the flow of tubal secretions. Proper density of ciliated cells is required to avoid ectopic pregnancy (Lyons et al., 2006). It has been demonstrated that fallopian tubes containing an ectopic pregnancy have a marked reduction in the number of ciliated cells in comparison with those of women with an intrauterine gestation (Vasquez et al., 1983; Lyons et al., 2006). Detailed ultrastructural analysis of hydrosalpinx of infertile women also demonstrates severe abnormalities in epithelial cells, including flattening of the epithelial layer and severe loss of ciliated cells (Ajonuma et al., 2005). Notably, ciliated cells and secretory cells are developmentally connected. A study using *in vivo* genetic cell lineage tracing in mice demonstrated that secretory epithelial cells not only self-renew, but also give rise to ciliated epithelial cells (Ghosh et al., 2017). LIF/LIFR signaling has been shown to alter the fate of epithelial cells during *Ct* infection (Kessler et al., 2019). The density of ciliated cells in organoids chronically infected with *Ct* is markedly reduced compared to non-infected controls (Kessler et al., 2019). This is likely mediated by LIF, as addition of recombinant LIF protein to non-infected organoids potently inhibits the frequency of ciliated cells (Kessler et al., 2019). The results from the organoid cultures provide strong experimental evidence supporting a potential role of LIF in epithelial transformation during *Ct* infection.

LIF is also recognized as a cell-autonomous molecule during intracellular viral infections caused by HIV (Patterson et al., 2002; Tjernlund et al., 2006) and HPV (Bay et al., 2011). We were unable to find any reports on LIF production caused by extracellular pathogens, such as *Neisseria gonorrhoeae*. Therefore, it is likely that LIF is produced preferentially, if not exclusively, upon intracellular infections for combating intracellular pathogens and assisting in tissue repair and epithelium homeostasis. While this is a protective response in nature, repeated *Ct* infections and/or persistent *Ct* infections may lead to aberrant expression of LIF and LIF-mediated alterations of the epithelium, which has been demonstrated in *in vitro* models of persistent chlamydial infections (Peters et al., 2005). Clinical observations indicate that most women with TFI and serological evidence of *Ct* infection lack a history of clinical PID (Brunham et al., 1985). Therefore, it is likely that the regulation of LIF and LIF-mediated responses are operating for long periods of time, during which, LIF levels are constantly being modified by surrounding proinflammatory cytokines and growth factors (**Table 3**). LIF expression is robustly induced by proinflammatory signals (e.g., LPS, IL-1 and TNF-α) (Wetzler et al., 1991; Ishimi et al., 1992; Hamilton et al., 1993; Wetzler et al., 1994; Arici et al., 1995; Perrier d'Hauterive et al., 2004) and signaling molecules active in tissue growth and development, including platelet-derived growth factor (PDGF) (Arici et al., 1995), epidermal growth factor (EGF) (Arici et al., 1995), human chorionic gonadotropin (hCG) (Perrier d'Hauterive et al., 2004), insulin-like growth factor (IGF) (Perrier d'Hauterive et al., 2004), and transforming growth factor-β (TGF-β) (Wetzler et al., 1991; Arici et al., 1995; Perrier d'Hauterive et al., 2004; Ruan et al., 2010; Ota et al., 2013). Many of these molecules are concurrently induced by *Ct* infection, which collectively amplify LIF expression and LIFR-mediated responses, and eventually lead to a marked reduction in ciliated epithelial cells and an increase of secretory epithelial cells in the fallopian tube. In comparison, IFN-γ potently supresses LIF expression in endometrial epithelial cells and stromal cells *in vitro (* Arici et al., 1995). Of note, IL-4 is a typical type 2 cytokine that regulates LIF in a cell type-dependent manner. IL-4 downregulates LIF in cultured bone marrow stromal cells, synovial fibroblasts and liver myofibroblasts (Wetzler et al., 1994; Denizot et al., 1999), whereas it upregulates LIF secretion in type 2 helper T-cells (Piccinni et al., 1998). Given that IFN-γ and IL-4 are primarily produced by T cells, it is possible that LIF production may diminish upon the establishment of adaptive immune responses. While LIF has been shown to promote regulatory T cells and inhibit the differentiation of type 17 helper T-cells (Metcalfe, 2011), it is unclear how T cell responses are regulated by prolonged LIF production during *Ct* infection. Although only limited knowledge is available, the crosstalk between LIF and T cells is likely an integral part of *Ct* pathogenesis and additional studies are warranted.

**Table 3:** Regulation of LIF expression.

Factor	Effect	Citation
p53	p53-knockout female mice show impaired fertility and reduced basal and induced LIF expression in uteri.	Reference Hu et al., 2007
Estrogen/Estradiol (E2)	E2 injections induces LIF expression in uterine tissues following E2 injections	References Chen et al., 2000; Ding et al., 2008; Liang et al., 2014; Yoo et al., 2019; Polim et al., 2022
Progesterone (P4)	P4 induces LIF expression in bovine endometrium epithelial cells *in vitro*	Reference Feng et al., 2022
Platelet derived growth factor	Upregulates LIF in human endometrium epithelial cells and stromal cells *in vitro*	Reference Arici et al., 1995
epidermal growth factor (EGF)	Upregulates LIF in human endometrium epithelial cells and stromal cells *in vitro*	Reference Arici et al., 1995
Chorionic gonadotropin	Upregulates LIF in human endometrium epithelial cells and stromal cells *in vitro*	Reference Peerier d'Hauterive et al., 2004
Insulin-like growth factor	Upregulates LIF in human endometrium epithelial cells and stromal cells *in vitro*	Reference Peerier d'Hauterive et al., 2004
TGF-β	Upregulates LIF in human endometrium epithelial cells and stromal cells, bone marrow stromal cells, and murine osteoblast cells *in vitro*	References Ruan et al., 2010; Wetzler et al., 1991; Arici et al., 1995; Perrier d'Hauterive et al., 2004; Ota et al., 2013
IL-1	Upregulates LIF in human endometrium epithelial cells and stromal cells, bone marrow stromal cells, osteoblasts and synovial fibroblasts *in vitro*	References Wetzler et al., 1991; Ishimi et al., 1992; Hamilton et al., 1993; Wetzler et al., 1994; Arici et al., 1995
TNF	Upregulates LIF in human endometrium epithelial cells and stromal cells, bone marrow stromal cells, osteoblasts and synovial fibroblasts *in vitro*	References Wetzler et al., 1991; Ishimi et al., 1992; Hamilton et al., 1993; Wetzler et al., 1994; Arici et al., 1995
LPS/Endotoxin	Upregulates LIF in osteoblasts *in vitro*	Reference Ishimi et al., 1992
IFN-ɤ	Inhibits LIF in human endometrium epithelial cells and stromal cells *in vitro*	Reference Arici et al., 1995

Based on the collective evidence discussed above, it is appealing to suggest that the LIF-mediated reduction in ciliated epithelial cell density is a key process of *Ct* pathogenesis, which can lead to reduced opportunities for fertilization and increased risk of ectopic pregnancy. Although it remains to be demonstrated experimentally, increased secretory cell density may lead to over-production and accumulation of fluids inside the fallopian tube, a characteristic feature of hydrosalpinx (Ng and Cheong, 2019). Consistent with this notion, LIF is found to be expressed in human fallopian tubes (Keltz et al., 1996) and is markedly elevated in chronically inflamed fallopian tubes (Ji et al., 2009). Furthermore, ectopic pregnancies are mostly associated with intracellular infections caused by *Ct* and *Mycoplasma genitalium*, and less frequently linked to extracellular pathogens like *Neisseria gonorrhoeae* (Ashshi et al., 2015; Refaat et al., 2016), despite its ability to cause a similar spectrum of pelvic inflammatory diseases (Xu and Gray-Owen, 2021).

The Human Protein Atlas dataset shows that high expression of LIF is a poor prognostic marker for human cervical cancer (Human Protein Atlas, 2022), for which HPV and *Ct* are well-known risk factors (Zhu et al., 2016; Liao et al., 2022). *Ct* infection is also a risk factor for ovarian cancer (Hosseininasab-Nodoushan et al., 2021). While HPV promotes cervical cancer *via* oncogenic transformation (Baedyananda et al., 2022), *Ct* may promote cervical cancer and ovarian cancer *via* aberrant LIF signaling, which has been shown to regulate multiple hallmarks of cancer, including proliferation, metastasis and chemoresistance (Jones and Jenkins, 2018; Jorgensen and de la Puente, 2022). LIF also has a significant role in enriching and maintaining cancer stem cells, epithelial to mesenchymal transition, de-differentiation, and re-differentiation of cancer cells (Halder et al., 2022).

## Conclusion remarks

**Figure 4:**
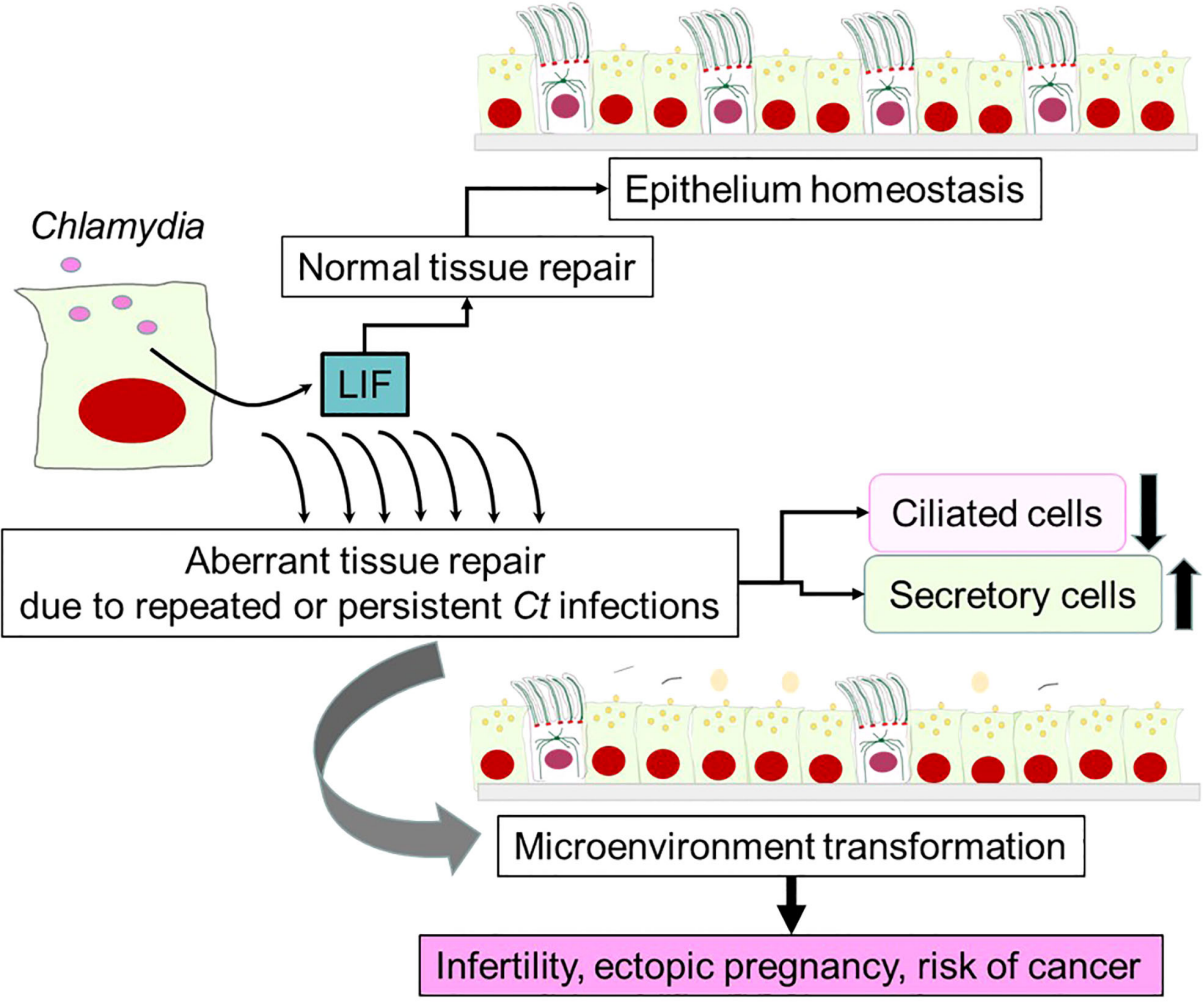
A potential role of LIF in *Chlamydia* pathogenesis. LIF is produced by epithelial cells upon *Ct* infection, which is required for maintaining epithelium homeostasis and normal tissue repair *via* autocrine LIF/LIFR signaling pathways. This would occur in many *Ct-*infected women without leaving serious complications. However, aberrant tissue repair is triggered by repeated or persistent infections, which results in marked reductions of ciliated epithelial cells and increases in secretory cells over time. The alteration in the cellular composition creates a microenvironment in fallopian tubes that may promote infertility, ectopic pregnancy and cancer.

In conclusion, available evidence supports a novel aspect of *Ct* pathogenesis controlled by the pleiotropic cytokine LIF (**Figure 4**). Despite the intended purpose of LIF production as a part of the host defense against intracellular *Ct* infection and host protective tissue healing, LIF-mediated signaling, particularly prolonged strong signaling, gradually transforms the microenvironment of the fallopian tube by diminishing the density of ciliated epithelial cells and increasing the population of less differentiated secretory epithelial cells. This harmful transformation of epithelium might be a key process leading to an enhanced risk of infertility, ectopic pregnancy and cancer.

## Author contributions

All authors listed have made a substantial, direct, and intellectual contribution to the work and approved it for publication.

## Funding

The study is supported by operating grant of the Canadian Institute of Health Research (CIHR) (Grant number: 201803PJT-159700) to JW. KW is supported by a Dalhousie Medical Research Foundation (DMRF) – Infection, Immunity, Inflammation & Vaccinology (I3V) Graduate Studentship.

## Conflict of interest

The authors declare that the research was conducted in the absence of any commercial or financial relationships that could be construed as a potential conflict of interest.

## Publisher’s note

All claims expressed in this article are solely those of the authors and do not necessarily represent those of their affiliated organizations, or those of the publisher, the editors and the reviewers. Any product that may be evaluated in this article, or claim that may be made by its manufacturer, is not guaranteed or endorsed by the publisher.
